# Supplementary material for: Twenty-Three Months Repetitive Transcranial Magnetic Stimulation of the Primary Motor Cortex for Refractory Trigeminal Neuralgia: A Single-Case Study
Source: Life (Basel). 2023 Jan 2;13(1):126. doi: 10.3390/life13010126 (PMC9866023; doi:10.3390/life13010126)
Supplement: Supplementary file 1 [file life-13-00126-s001.zip › life-2079853-supplementary.pdf]

Table S1. Results of the Wilcoxon matched-pairs signed rank test performed on the baseline and intervention phase pain scores.

**Week no.**			**Week no.**			**Week no.**		
	Z	*p*-value		*Z*	*p*-value		*Z*	*p*-value
2	-1.913	.056	29	-2.388	.017*	56	-2.375	.018*
3	-2.371	.018*	30	-2.388	.017*	57	-2.375	.018*
4	-2.388	.017*	31	-2.388	.017*	58	-2.371	.018*
5	-2.366	.018*	32	-2.375	.018*	59	-2.371	.018*
6	-2.201	.028*	33	-2.371	.018*	60	-2.384	.017*
7	-2.375	.018*	34	-2.375	.018*	61	-2.388	.017*
8	-2.366	.018*	35	-2.428	.015*	62	-2.366	.018*
9	-2.375	.018*	36	-0.594	.553	63	-0.314	.753
10	-2.201	.028*	37	-2.371	.018*	64	-2.388	.017*
11	-2.388	.017*	38	-2.384	.017*	65	-2.366	.018*
12	-2.388	.017*	39	-2.388	.017*	66	-2.388	.017*
13	-2.375	.018*	40	-2.371	.018*	67	-2.375	.018*
14	-2.371	.018*	41	-2.375	.018*	68	-2.375	.018*
15	-2.375	.018*	42	-2.375	.018*	69	-2.366	.018*
16	-2.375	.018*	43	-2.375	.018*	70	-2.388	.017*
17	-2.375	.018*	44	-2.375	.018*	71	-2.371	.018*
18	-2.388	.017*	45	-2.375	.018*	72	-2.388	.017*
19	-2.371	.018*	46	-2.388	.017*	73	-2.388	.017*
20	-2.401	.018*	47	-2.375	.018*	74	-2.388	.017*
21	-2.375	.018*	48	-2.371	.018*	75	-2.388	.017*
22	-2.375	.018*	49	-2.371	.018*	76	-2.375	.018*
23	-2.375	.018*	50	-2.384	.017*	77	-2.388	.017*
24	-2.375	.018*	51	-2.371	.018*	78	-2.032	.042*
25	-2.375	.018*	52	-2.371	.018*	79	-2.371	.018*
26	-2.392	.017*	53	-2.384	.017*	80	-2.375	.018*
27	-2.388	.017*	54	-2.375	.018*	81	-2.375	.018*
28	-2.388	.017*	55	-2.371	.018*			

* = indicate significant results ($p < 0.05$).
